# Genetic susceptibility of COVID-19: a systematic review of current evidence

**DOI:** 10.1186/s40001-021-00516-8

**Published:** 2021-05-20

**Authors:** SeyedAhmad SeyedAlinaghi, Mohammad Mehrtak, Mehrzad MohsseniPour, Pegah Mirzapour, Alireza Barzegary, Pedram Habibi, Banafsheh Moradmand-Badie, Amir Masoud Afsahi, Amirali Karimi, Mohammad Heydari, Esmaeil Mehraeen, Omid Dadras, Jean-Marc Sabatier, Fabricio Voltarelli

**Affiliations:** 1 Iranian Research Center for HIV/AIDS, Iranian Institute for Reduction of High Risk Behaviors, Tehran University of Medical Sciences, Tehran, Iran; 2 Healthcare Services Management, School of Medicine and Allied Medical Sciences, Ardabil University of Medical Sciences, Ardabil, Iran; 3 School of Medicine, Islamic Azad University, Tehran, Iran; 4 Black Dog Institute, University of New South Wales, Sydney, Australia; 5 Department of Radiology, School of Medicine, University of California, San Diego (UCSD), San Diego, CA USA; 6 School of Medicine, Tehran University of Medical Sciences, Tehran, Iran; 7 Department of Health Information Technology, Khalkhal University of Medical Sciences, 1419733141 Khalkhal, Iran; 8 Department of Global Health and Socioepidemiology, Graduate School of Medicine, Kyoto University, Kyoto, Japan; 9 Institut deNeuro-Physiopathologie (INP), UMR 7051, Faculté de Pharmacie, Université Aix-Marseille, 27 Bd Jean Moulin, 13385 Marseille Cedex, France; 10 Graduation Program of Health Sciences, Faculty of Medicine, Federal University of Mato Grosso, Cuiabá, Brazil

**Keywords:** Genetic susceptibility, Genetic vulnerability, Genetic probability, COVID-19, SARS-CoV-2

## Abstract

**Introduction:**

While COVID-19 pandemic continues to spread worldwide, researchers have linked patterns of traits to poor disease outcomes. Risk factors for COVID-19 include asthma, elderly age, being pregnant, having any underlying diseases such as cardiovascular disease, diabetes, obesity, and experiencing lifelong systemic racism. Recently, connections to certain genes have also been found, although the susceptibility has not yet been established. We aimed to investigate the available evidence for the genetic susceptibility to COVID-19.

**Methods:**

This study was a systematic review of current evidence to investigate the genetic susceptibility of COVID-19. By systematic search and utilizing the keywords in the online databases including Scopus, PubMed, Web of Science, and Science Direct, we retrieved all the related papers and reports published in English from December 2019 to September 2020.

**Results:**

According to the findings, COVID-19 uses the angiotensin-converting enzyme 2 (ACE2) receptor for cell entry. Previous studies have shown that people with ACE2 polymorphism who have type 2 transmembrane serine proteases (TMPRSS2) are at high risk of SARS-CoV-2 infection. Also, two studies have shown that males are more likely to become infected with SARS-CoV-2 than females. Besides, research has also shown that patients possessing HLA-B*15:03 genotype may become immune to the infection.

**Conclusion:**

Combing through the genome, several genes related to immune system’s response were related to the severity and susceptibility to the COVID-19. In conclusion, a correlation was found between the ACE2 levels and the susceptibility to SARS-CoV-2 infection.

## Introduction

Coronavirus Disease-2019 (COVID-19) started its devastating trajectory into a global pandemic in Wuhan, China [1–6]. The World Health Organization (WHO) declared this disease a “public health emergency of international concern” on January 30, 2020 [7–9]. Until April 1, the WHO reports a total number of 127,877,462 confirmed cases and 2,796,561 deaths [10]. Although capable of causing severe pneumonia, severe acute respiratory syndrome-Coronavirus-2 (SARS-CoV-2) can also involve various organs and cause physical symptoms, as well as psychological damages [11–14].

SARS-CoV-2 utilizes molecules to enter the cells, such as angiotensin-converting enzyme 2 (ACE2) to attach to the receptor-binding domain (RBD) and type 2 transmembrane serine proteases (TMPRSS2) to cleave the spike (S) protein [15–17]. SARS-CoV-2 S-protein cleavage by TMPRSS2 initiates viral entry and also helps the virus escape the immune system [15]. The hidden receptor-binding domain attached to ACE2 enhances immune evasion by weakening the immune surveillance. SARS-CoV-2 S-protein cleavage by TMPRSS2 is also responsible for immune escape besides initiating the viral entry [15]. Hence, genetic differences among such molecules responsible for cellular entry might alter the observed responses among different individuals [18, 19]. Various cells in the body express ACE2 to different extents; and therefore, numerous organs of the body can be invaded by the virus, such as the lungs and the heart [18, 20–22]. Additionally, cell entry is reinforced in the presence of TMPRSS2 in ACE2-positive cells by cleaving the viral Spike protein, which can result in its activation for membrane fusion [23–25]. There are reports that not only genetic differences can cause certain races and populations to be more affected by this virus, but also they might contribute to gender-specific differences [26]. Higher testosterone levels enhance TMPRSS2 levels and might cause higher disease susceptibility in males [27, 28]. To make matters even more important, the differences in severity observed in children compared to older adults may be in parts due to the different expressions of genetic components, such as TMPRSS2 [29].

As mentioned above, various genetic patterns might alter the risk of infection, viral entry, immune response to the virus, COVID-19 severity, and death. Therefore, we aim to review the published literature on the genetic aspects of susceptibility to COVID-19 and discuss the above-mentioned statements. Recognizing these points might help us determine at-risk groups and take specific measures to support them.

## Methods

This study aims to review the current evidence of genetic susceptibility to COVID-19 carried out in September 2020. The authors studied the probable genetic vulnerability to novel Coronavirus (COVID-19). In this study, the preferred reporting items for systematic reviews and meta-analyses (PRISMA) checklist is used as a criterion to ensure the accuracy and reliability of the results.

### Data sources

By systematic search and utilizing the keywords in the online databases including Scopus, PubMed, Web of Science, and Science Direct, we retrieved all related papers and reports published in English from December 2019 to September 2020. To convey our search strategy, we considered different combinations of keywords in the following orders:

“COVID-19” or “SARS-CoV-2” or “Novel Coronavirus” or “2019-nCoV” or “Coronavirus” (title/abstract).
“Genetic susceptibility” or “Genetic vulnerability” or “Genetic probability” (title/abstract).
(A) and (B).

### Study selection

Two independent investigators screened and selected the studies with the most relevant titles and abstracts. Then, the full text of the extracted papers was reviewed, and based on the eligibility criteria, the most relevant papers were selected. The peer-reviewed original papers published in English that satisfied the eligibility criteria were considered in the final report.

Our exclusion criteria for this study, whereas the following measures:

- Papers focusing on non-human studies including in vitro observations or researches concentrating on animal experiments, or discussing COVID-19 without a satisfying detail, and papers without referring to the keywords of this study.
- Papers whose full texts were not accessible.
- Any results were suspicious of duplication in databases.

### Data extraction

After the summarization process, data including type of article (e.g., case reports), information of authors, country of origin, publication date, sample size, gender, age, and genetic susceptibility data were transferred into an organized sheet. Two independent investigators gathered this information. Finally, the other authors cross-checked all the selected papers to make sure no overlap or duplications exist in the content.

### Quality assessment

As previously mentioned, we considered the preferred reporting items for systematic reviews and meta-analyses (PRISMA) checklist to ensure the quality and authenticity of selected studies. The quality and consistency of the articles and the probable risk of bias were evaluated by two independent researchers. A third independent researcher was recruited to resolve the issue in any situation where differences in thoughts were encountered. In the end, the full text of selected articles was read and the significant findings of each article were extracted.

## Results

**Fig. 1:**
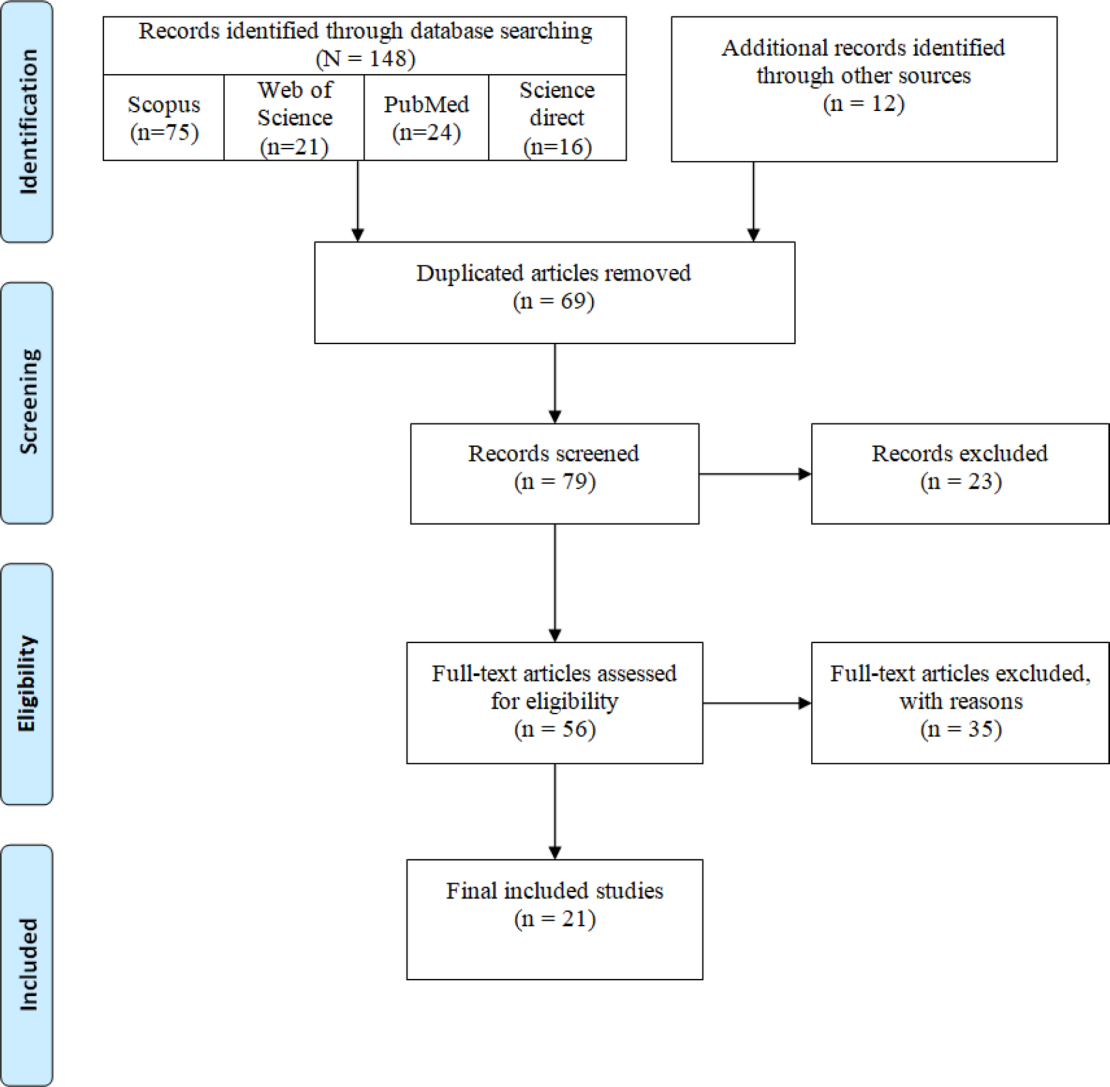
Flow diagram of the selection process of articles identified

Using the described search strategy, we retrieved 148 full-text articles. After an initial review of retrieved resources, 69 duplicates were removed, and the title and abstract of the remaining 79 articles were reviewed. Applying the selection criteria, 58 articles were excluded, and only 21 articles met the inclusion criteria and were included in the final review (Fig. 1).

According to the findings, COVID-19 uses the ACE2 receptor for cell entry. A review of articles showed individuals with ACE2 polymorphism along with TMPRSS2 are more vulnerable to COVID-19. Two studies have shown that males are more likely to become infected with SARS-CoV-2 than females. Besides, research have also shown that patients possessing HLA-B*15:03 genotype may become immune to the infection (Table 1).

**Table 1:** Identified genetic susceptibility for COVID-19

ID	The first author (reference)	Type of study	Country	Study population	Risk factors	Important findings
1	Abhari and Kawwass [30]	Review	USA	112 women with normal endometrial pathology	ACE2, TMPRSS2, TMPRSS4, cathepsin B and L (CTSB and CTSL, respectively), FURIN, MX dynamin-like GTPase 1 (MX1), Basigin (BSG) genes	A positive correlation in ACE2 and TMPRSS4 expression has been found during the early secretory phase, beside a weak but positive correlation in ACE2, TMPRSS4, and CTSL expression during the window of implantation. They also reported that the expression of most viral genes including ACE2, TMPRSS4, CTSL, and CTSB at different stages of the menstrual cycle increases with age. Their findings suggest that older women may be more vulnerable to SARS-CoV-2 endometrial infection
2	Cao et al. [31]	Letter to the editor	China	COVID-19 patients from different populations	ACE2 gene	People living in East Asia have much higher AFs in the eQTL variants associated with higher ACE2 expression in tissues. It may result in a different susceptibility or response to SARS-CoV-2 indifferent populations under similar conditions
3	Casanova et al. [32]	Original research	USA	COVID-19 patients	IFN-γ, IL-17A/F genes	While known genetic factors are absent in determining human susceptibility to other Coronaviruses, influenza seems to provide the best comparison. Although the threshold levels of type I and/or III IFN for immunity against SARS-CoV-2 might be similar to those for the 1918 influenza virus, it might be higher than those for seasonal influenza
4	Debnath et al. [33]	Review	India	Covid-19 patients	ACE2, HLA, cytokine storm, TLR genes, complement genes	Numerous studies show the important role of genes coding ACE2, HLA, cytokine, TLR, and complement components in COVID-19. The distribution of many of these genes varies geographically or demographically and therefore causes different susceptibility and/or resistance to different viral diseases
5	Devaux et al. [34]	Review	France	COVID-19 patients	ACE2 gene	ACE2 protein at the surface of lung alveolar epithelial cells increases the level of vulnerability to SARS-CoV-2 infection. They assumed that the ACE2 levels correlate with susceptibility to SARS-CoV-2 infection. It should be considered that men have a higher ACE2 expression in the lung than women. In addition, Asian people express a higher level of ACE2 than Caucasian and African-American people
6	Fakhouri et al. [35]	Review	USA	COVID-19 patients	ACE2 gene	ACE levels are genetically controlled. People with ACE gene polymorphisms are more likely to become infected by the severe inflammation induced by COVID-19
7	Fujikura et al. [36]	Research	Japan	Data from 5 population—sequencing projects	ACE2 gene, TMPRSS2 gene	SNVs in ACE2 and TMPRSS2 are mostly are, population-specific, and harmful; however, some very rare SNVs might explain different susceptibility to SARS-CoV-2
8	Gemmati et al. [37]	Hypothesis	Italy	COVID-19 patients	ACE 2 gene, TMPRSS2 gene	Males are more likely to become infected with SARS-CoV-2 than females. A strong relation found between SARS-CoV-2 and human ACE 2 receptor
9	Giudicessi et al. [38]	Creative concepts	USA	African-Americans	A potentially pro-arrhythmic common variant, p.Ser1103-Tyr-SCN5A, present in 1 out of 13 individuals of African descent has the potential to increase the risk of drug- and hypoxia-induced ventricular arrhythmias/sudden cardiac death	They showed that the common ion channel variants p.Asp85Asn-KCNE1 and p.Ser1103TyrSCN5A confer an increased risk of DI-LQTS (drug-induced long QT syndrome) and DI-SCD (Drug-induced sudden cardiac death)
10	Godri et al. [39]	Review	USA	COVID-19 patients	Polymorphisms in ACE2 gene. Polymorphisms of cellular proteases along with furin and TMPRSS2. HLA-B*46:01 gene product (suggesting individuals with this allele may be more vulnerable to COVID-19)	HLA-B*15:03 (patients possessing HLA-B*15:03 genotype may become immune to the infectious COVID-19)
11	Hou et al. [40]	Correspondence	USA	COVID-19 patients	1. ACE2 polymorphisms were more likely to be associated with cardiovascular and pulmonary conditions by altering the angiotensinogen-ACE2 interactions, such as p.Arg514-Gly in the African/African-American population	Developmental regulation of TMPRSS2 may make the infants and children immune to COVID-19. Thus, research should investigate the relationship between age and polymorphisms for TMPRSS2
					2. We found 6 germline deleterious variants (p.Val160Met, p.Gly181Arg, p.Arg240Cys, p.Gly259Ser, p.Pro335Leu, and p.Gly432Ala) in the TMPRSS2 coding region	
12	Junejo et al. [41]	Review	Pakistan/Turkey	COVID-19 patients		Loss of ACE2 function is related to acute lung injury because the ACE2 downregulation can result in RAS dysfunction, and endorse the inflammation that causes vascular permeability
13	Kachuri et al. [42]	Research study	USA	COVID-19 patients	ACE2 gene	Sars-Cov-2 uses the ACE2 receptor for cell entry
14	LoPresti et al. [43]	Review	USA	COVID-19 patients	ACE2 gene, TMPRSS2 gene, HLA-B*46:01	HLA-B*15:03 may be protective
15	Sanchez-Gonzalez et al. [44]	Report	USA	COVID-19 patients	Telomeres	In addition to people with immune senescence and dysregulation, SARS-CoV-2 can stimulate an HP-like severe immune reaction, especially in genetically vulnerable individuals. Telomeres typically shorten as the person gets older, but different rates of shortening may allow for measuring chronological versus biological aging. Besides, there is a strong connection between SARS-CoV-2 severe cases and the age of patients. There should be some researches on the association between telomere length and SARS-CoV-2, especially in younger patients with severe disease
16	Sheikh et al. [45]	Report	India	Covid-19 patients	ACE2	–
17	Sironi et al. [46]	Review	USA	COVID-19 patients	ACE2	S1 spike proteins of SARS-CoV and SARS-CoV-2 connect to ACE2 on the surface of the cell, causing the cleavage of the vasoconstrictor angiotensin II and countering the activity of ACE
					TMPRSS2	Lopera et al. [47] have reported a relation between a PheWAS of 178 quantitative phenotypes, including cytokine and cardio-metabolic markers which are not specific SARS-CoV-2 infection markers, and ACE2 and TMPRSS2 variation
18	Strafella et al. [48]	Cohort	Italy	Italian population	ACE2	They identified five SNVs in the Italian cohort: rs35803318, rs41303171, rs774469453, rs773676270, and rs2285666. Among them, rs35803318 and rs2285666 had a significantly different frequency distribution in the Italian people concerning worldwide population. The eQTLs analysis of ACE2 revealed a high distribution of eQTL variants in different brain tissues. They suggested that there may be a connection between ACE2 genetic variability and the neurological complications in patients with COVID-19
19	Torre-Fuentes et al. [49]	Cohort	Spain	138 individuals: 52 patients with MS and 86 unaffected family members	ACE2 gene, TMPRSS2 gene	Eleven variants were detected, which three of them were missense: rs75603675, rs12329760, and rs200291871, and eight of them were synonymous variants: rs2298659, rs61735792, rs61735789, rs61735794, rs141788162, rs17854725, rs3787950, andrs142750000, and for the remaining TMPRSS2, there weren’t any significant differences between individuals with and without SARS-CoV-2
20	Thüsen and Eerden [50]	Review	Netherlands	COVID-19 patients	ACE2 gene, TMPRSS2 gene	Genetics may also play a role through polymorphisms of genes that encode for two types of proteins. First, proteins that are exploited by SARS-CoV-2, such as the highly conserved ACE2, which uses for docking and cellular entry in respiratory cells, as it was also the case for SARS-CoV. Second, proteins that protect against the effects of the virus (such as surfactant proteins, but also ACE2 itself). As an X-linked phenotype, interaction-booster and interaction-inhibitor variants of ACE2 can have a more definite effect in males than females and could play a role in a higher mortality rate in males. This can be the result of up to ∼70% of death caused by SARS-CoV2, SARS-CoV or MERS-CoV, in addition to a likely higher rate of risk factors (e.g., smoking) and a possibly different immune response in males. Other factors may also contribute. For example, transmembrane protease serine 2 (TMPRSS2), and its variants and expression have been linked to differences in COVID-19 severity
21	Vuitton et al. [51]	Editorial	France	COVID-19 patients	Immune complexes (ICs), ACE2	In SARS-CoV-2 infection, the virus binds to ACE2, which is an enzymatic inhibitor of angiotensin II, able to modify the local microenvironment of ICs in vessels and alveoli. This mechanism could be a stimulant for IC-related endotheliitis

## Discussion

Consistent with the general conclusion, there are several genetic patterns of susceptibility for COVID-19, including: ACE2, TMPRSS2, Cathepsin, FURIN, MX dynamin-like GTPase 1 (MX1), Basigin (BSG), IFN-γ, IL-17A/F, HLA, cytokine storm, TLR genes, p.Ser1103, Tyr-SCN5A, p.Arg514Gly, p.Val160Met, p.Gly181Arg, p.Arg240Cys, p.Gly259Ser, p.Pro335Leu, p.Gly432Ala, Telomeres, and immune complexes (ICs) [18, 19, 30, 32, 33, 38–40, 43, 44, 51]. Our review demonstrated that genetic risk factors can affect the treatment and prevention of COVID-19.

ACE2 and TMPRSS2 DNA polymorphisms have been shown to be strongly associated with the susceptibility, severity, and clinical outcomes of COVID-19 [18, 19]. Structural analysis of the binding interface between SARS-CoV-2 RBD and human ACE2 shows a strong network of polar contacts [46]. SARS-CoV-2 RBD mediates these polar interactions through Lys417, Gly446, Tyr449, Asn487, Gln493, Gln498, Thr500, Asn501, Gly502 and Tyr505 with ACE2 [46]. Various gender and age groups have significantly different susceptibilities to infection and mortality rates [52]. Old age males with comorbidities are considered the most vulnerable groups [27, 28, 30, 52]. SARS-CoV-2 has a very strong interaction with the ACE2 receptor, and it has an important role in cell entry together with TMPRSS2 [18, 19, 52–57]. Males are at higher risk of severe COVID-19 compared to women and are responsible for 60% of the deaths [30, 52]. The ACE2 expression rate is higher in females than males, single nucleotide polymorphisms (SNPs) within the TMPRSS2 gene (21q22.3) can also have a greater role in the general population (rs2070788, rs7364083, rs9974589) and in a sex-oriented perspective (rs8134378) assuming that higher expression in males might favor virus membrane fusion [26, 37].

ACE2 is a receptor for SARS-CoV-2. Also, it can facilitate the post-infection downstream processes including inflammatory responses [33]. ACE2 receptors are ubiquitous and widely expressed in the heart, vessels, gut, lung, kidney, testis, and brain [20, 22, 58]. SARS-CoV-2 enters into the cell through binding of the viral S-protein to the ACE2 receptors [15, 17]. The ACE2 receptors diminish the unfavorable effects of angiotensin II in several ways [58]. First, they can degrade angiotensin II and reduce its deleterious effect, and second, by generating angiotensin (1–7) they perform counter-regulatory effects to those of angiotensin II [59]. Several conditions including older age, diabetes, hypertension, and heart diseases like heart failure can lead to ACE2 deficiency [58]. Given the above information, ACE2 deficiency is supposed to play a pivotal role in the pathogenesis of SARS-CoV-2 infection which favors the progression of inflammatory and thrombotic processes [58].

As mentioned earlier, binding SARS-CoV-2 to ACE2 is facilitated by a RBD located within the S-protein of the virus. There are 25 different ACE2 gene variants in the Leiden open variation database (LOVD) [37]. Two specific ACE2 alleles (i.e., rs73635825 and rs143936283) showed a low binding affinity of the entire SARS*-*CoV*-*2 S-protein, so viral attachment decreased and potential resistance to infection was reported [37]. According to recent studies, genetic polymorphisms in the ACE2 gene were detected in populations, which modulate the SARS*-*CoV*-*2 affinity spike protein and worsen pulmonary and systemic injury by accelerating lung damage in COVID-19 patients, which enhanced vasoconstriction, inflammation, oxidation, coagulation, capillary permeability, fibrosis, and apoptosis in the alveolar cells [37, 60]. Single-nucleotide polymorphism (SNPs) in ACE2 is associated with different diseases. Therefore, SNPs can impact gene expression, which might influence disease severity. Moreover, COVID-19 is referred to the state of cytokine storm which results in an excessive release of cytokines that lead to multiple organ damage. Such response cytokine storm was showed in ACE2-positive cells. Thus, differences in response to COVID-19 associated with genetic polymorphism within the genes responsible for the synthesis of cytokines and pro-inflammatory mediators along with ACE2 [61].

Male express higher ACE2 levels in their type II pneumocytes; and therefore, their lung samples may express threefold higher levels of ACE2 compared to the females [53–57]. In addition, the ACE2 expression in Asian populations is higher than African-American and Caucasian populations [31, 34]. As the evidence showed, the conversion of angiotensin II to angiotensin (1–7) by ACE2 was higher in males than females, indicating over expression of ACE2 in males [54]. As ACE2 is encoded by a gene located on the X chromosome and males express more ACE2 than females it could be considered that depending on the allele expressed by females, they could be considered of lower sensitivity against the most severe adverse effects of the infection [54].

As thoroughly discussed above, ACE2 protein at the surface of lung alveolar epithelial cells allows infection of the respiratory tract with SARS-CoV-2 [54]. In the lung, SARS-CoV-2 mainly infects alveolar epithelial cells, resulting in respiratory symptoms, and SARS-CoV infects mainly pneumocytes and macrophages. ACE2 expression protects from lung injury and is down-regulated by SARS-S. The expression of ACE2 also manifests itself beyond the lung. Since ACE2 is a functional receptor for SARS-CoV-2, researchers started to carefully consider the safety and potential effects of antihypertension therapy with ACE inhibitors or angiotensin-receptor blockers in patients with COVID-19. Besides, diabetes mellitus and hypertension are known as the most common comorbidities in COVID-19; both these conditions are controlled by ACE2. Earlier researchers were concerned on whether patients with COVID-19 and hypertension who are taking an ACE inhibitor (ACEI) or angiotensin-receptor blocker (ARB) medication should seek another antihypertensive drug [11, 41]. However, novel studies refuted the association between ACEI/ARB use and the COVID-19 disease severity; and therefore, physicians should not have concerns regarding prescribing these medications during the pandemic [62, 63].

On the other hand, a TMPRSS2 inhibitor can be a treatment option by blocking the cell entry, since an inhibitor of TMPRSS2 can block SARS-CoV-2 infection of lung cells [18, 41].

ACE2 can cause SARS-CoV-2 infection in the host cells, while also causing damage to the myocardium; however, the specific mechanisms have not yet been precisely discovered. Some studies indicated that ACE2 polymorphisms were in association with cardiovascular conditions by modifying the angiotensinogen–ACE2 interactions, asp.Arg514Gly in the African/African-American people. Particularly, 54% (33/61) and 39% (24/61) of deleterious variants in ACE2 appear in non-Finnish European (EUR) and African/African-American (AFR) populations, respectively. One of the reasons for the difference in genetic susceptibility to COVID-19 on one hand, and risk factors like high-risk male patients with cancer, on the other hand, can be the presence of a high number of polymorphisms in TMPRSS2 [e.g., p.Val160Met (rs12329760) which is an expression quantitative trait locus (eQTL)]. Moreover, the comparative genetic analyses implied that ACE2 genomic variants play critical roles in susceptibilities to COVID-19 and its associated cardiovascular conditions by altering the AGT-ACE2 pathway. Also, the developmental regulation of TMPRSS2 may make infants and children immune from COVID-19. The prevalent polymorphisms in ACE2 or TMPRSS2 lead to the use of personalized treatments (i.e., hydroxychloroquine and camostat) for COVID-19. However, the evidence refuted the effectiveness of these treatments and ruled out their use as logical treatments [64]. Also TMPRSS2 variants and resulting expression influence COVID-19 severity [26, 65]. For example, endometrium carries low susceptibility to COVID-19 due to the low expression of TMPRSS2 and ACE2. However, expression of the genes related to SARS-CoV-2 infection varies with menstrual cycle phase and age, with older women expressing higher ACE2 levels [30].

Last but not least, the similarity of the chemokine receptor protein encoded by the CCR5 gene for HIV has been compared to ACE2 for SARS-CoV. The target of HIV infection is CCR5 gene, and genetic variations in CCR5 can result in the different rates of this infection. ACE2 can play the role of an efficient or inefficient receptor for SARS-CoV if the amino acid remains are altered. Several ACE2 variants could have an important influence on the association between ACE2 and S-protein in SARS-CoV. Therefore, this gene variation can justify the gender differences and different susceptibility and disease severity to SARS-CoV infection in different racial groups [36].

In addition, some evidence indicates that the entry of SARS-CoV-2 into the cell is facilitated by the polymorphisms of the cellular proteases in the presence of furin [65]. The furin can be connected to the SARS-CoV-2, and the S-protein might be cleaved by TMPSS2 with the collaboration of furin. This function results in the entry of the virus into the respiratory tract and also in an increased risk of infection. Despite the aforementioned procedures, an association between furin and COVID-19 has not yet been discovered [49].

## Conclusion

Combing through the genome, a relation has been found between the severity and susceptibility of COVID-19 and some genes associated with the immune system’s response. In conclusion, a correlation was found between the ACE2 levels and the susceptibility to SARS-CoV-2 infection. Therefore, in SARS-CoV-2, as well as SARS-CoV, ACE2 has been recognized as a functional receptor. ACE2 seems to be crucial in outcomes of COVID-19, and the efficiency of ACE2 usage is a significant element of SARS-CoV transmissibility. SARS-2-S uses ACE2 (SARS-CoV receptor) to enter the cells and the serine protease TMPRSS2 for S-protein preparing to the entrance.

## Data Availability

The authors stated that all information provided in this article could be shared.

## Abbreviations

ACE2: Angiotensin-converting enzyme 2
AFR: African/African-American
BSG: Basigin
COVID-19: Coronavirus disease-2019
HIV: Human immunodeficiency virus
EUR: Non-Finnish European
PRISMA: Preferred reporting items for systematic reviews and meta-analyses
TMPRSS2: Type 2 transmembrane serine proteases
WHO: World Health Organization
